# The value of metabolic LncRNAs in predicting prognosis and immunotherapy efficacy of gastric cancer

**DOI:** 10.3389/fonc.2022.1019909

**Published:** 2023-01-04

**Authors:** Peizhun Du, Pengcheng Liu, Rajan Patel, Shiyu Chen, Cheng’en Hu, Guangjian Huang, Yi Liu

**Affiliations:** ^1^ Department of General Surgery, Huashan Hospital, Fudan University, Shanghai, China; ^2^ A1 Legend, Privia Health, Gaithersburg, MD, United States; ^3^ Department of Pathology, Huashan Hospital, Fudan University, Shanghai, China; ^4^ Department of Digestive Disease, Huashan Hospital, Fudan University, Shanghai, China

**Keywords:** lncRNA, gastric cancer, prognosis, metabolism, immune microenviroment

## Abstract

**Introduction:**

As a unique feature of malignant tumors, abnormal metabolism can regulate the immune microenvironment of tumors. However, the role of metabolic lncRNAs in predicting the prognosis and immunotherapy of gastric cancer (GC) has not been explored.

**Methods:**

We downloaded the metabolism-related genes from the GSEA website and identified the metabolic lncRNAs. Co-expression analysis and Lasso Cox regression analysis were utilized to construct the risk model. To value the reliability and sensitivity of the model, Kaplan–Meier analysis and receiver operating characteristic curves were applied. The immune checkpoints, immune cell infiltration and tumor mutation burden of low- and high-risk groups were compared. Tumor Immune Dysfunction and Exclusion (TIDE) score was conducted to evaluate the response of GC patients to immunotherapy.

**Results:**

Twenty-three metabolic lncRNAs related to the prognosis of GC were obtained. Three cluster patterns based on metabolic lncRNAs could distinguish GC patients with different overall survival time (OS) effectively (p<0.05). The risk score model established by seven metabolic lncRNAs was verified as an independent prognostic indicator for predicting the OS of GC. The AUC value of the risk model was higher than TNM staging. The high-risk patients were accompanied by significantly increased expression of immune checkpoint molecules (including PD-1, PD-L1 and CTLA4) and increased tumor tolerant immune cells, but significantly decreased tumor mutation burden (TMB). Consistently, TIDE values of low-risk patients were significantly lower than that of high-risk patients.

**Discussion:**

The metabolic lncRNAs risk model can reliably and independently predict the prognosis of GC. The feature that simultaneously map the immune status of tumor microenvironment and TMB gives risk model great potential to serve as an indicator of immunotherapy.

## Introduction

Gastric cancer (GC) is a common cancer worldwide and carries an incidence of over one million new cases in 2020 and an estimated 769,000 deaths, ranking fifth for incidence and fourth for mortality globally (1). The high mortality might be ascribed to the diversity of tumors, but the treatment is unitary. Immunotherapy showed promising efficacy in a subset of GC patients, and brought the dawn to the treatment of GC. More accurate classification and immunotherapy for suitable GC patients are urgently needed to solve the dilemma behind GC treatment and to improve the overall survival (OS) rate.

Tumor metabolism and immune environment have been the focus of research in recent years. It is now believed that the stimulation of the external environment and the abnormal regulation of oncogenes and tumor suppressor genes are the root causes of abnormal metabolism of tumor cells. Abnormal metabolism not only satisfies the needs of rapid tumor growth, but also has a far-reaching impact on tumor invasion, metastasis and therapy resistance (2, 3). Dysfunctional immune status in cancer microenvironment is a hallmark of cancer. The tumor can force the body to shift to a low immune reaction or low tolerance state through many ways, including interfering with the antigen presentation of dendritic cells, hindering the activation and immune response of T cells and abnormal expression of autoantigen, eventually producing a microenvironment conducive to its growth (4). Tumor immune escape is an important strategy of tumor survival, which has become a research hotspot in recent years (5).

With further research, it was found that the tumor metabolism and immunosuppression are not unrelated (6). During the process of rapid proliferation, tumor cells sample the surrounding microenvironment for nutrient locations, which can lead to changes in the immune microenvironment (7). For example, Warburg metabolism provides a cell extrinsic advantage to tumor cells, with accelerated exhaustion of extracellular glucose, rendering tumor infiltrating T cells dysfunctional (8). Metabolic changes in T cells would inhibit anti-tumor T effector cell response, induce Treg (T regulatory) cells response and mediate immunosuppression, thereby promoting tumor progression (9). The immune response of the body to the tumor is in a low-energy state, such that the tumor can escape immune surveillance and progress. Evidence suggested that long noncoding RNA (lncRNA) can modulate tumor metabolism and innate immunity by targeting various metabolic pathways in different ways, either through cis-regulation, antisense inhibition, interaction with proteins or interaction with microRNA (miRNA) sponges (10, 11).

LncRNA is a type of RNA that cannot encode protein. It was not considered to be of any value in the process of gene transcription and had no physiological function. With the development of new technology, research on lncRNA has been performed in more depth over the past few years. LncRNA plays the role of regulators in various biological processes of eukaryotes, and shows abnormal expression in a variety of malignant tumors. Their abnormal expression is closely related to the degree of tumor malignancy, including tumor growth, drug resistance, metastasis, immunity and metabolism. For instance, Li et al. found that lncRNA LIMIT locally targets GBPs, thereby forming a molecular cascade of LIMIT–GBP–HSF1–MHC to alter antitumour immunity and the efficacy of tumour immunotherapy (12). LncRNA lincNMR regulates nucleotide metabolism *via* interacting with YBX1 and regulating RRM2, TK1, and TYMS (13).

Currently, the methodology of repurposing used microarray data for expression profiling of ncRNAs (noncoding RNA) has been well established. For instance, Song et al. used a series of microarray datasets to build a resource of clinically relevant lncRNAs and found a tumor-specific prognostic lncRNA model in GC (14). However, whether metabolic lncRNAs can build an effective model to judge the prognosis and immunotherapy efficacy of tumors is unknown. In this study, we constructed a predictive model of GC with metabolic lncRNAs based on the Cancer Genome Atlas (TCGA) database. The performance of metabolic lncRNAs model in prognosis, immune microenvironment and immunotherapy of GC was investigated.

## Patients and methods

### Patients and samples

The gene expression profiles and the clinical characteristics of samples were downloaded from the TCGA database (https://gdc.nci.nih.gov) [15]. 375 GC tissues and 32 normal tissues derived by HIseq-FPKM (Fragments Per Kilobase Million) were enrolled in the study. The collected clinicopathological data included gender, age, TNM (tumor node metastasis) classification, survival status, TNM staging and survival outcomes. The downloaded raw data pre-procession and bioinformatics analyses were conducted using the R studio software. The clinicopathological features of GC patients were described in the **Supplementary File**.

### RNA sequence analysis of metabolism-related genes and lncRNAs

The list of metabolism-related genes was downloaded from the GSEA(Gene Set Enrichment Analysis) website (https://www.gsea-msigdb.org/gsea/index.jsp). Strawberry Perl was used to extract metabolism-related genes from TCGA-GC samples. We used the Wilcoxon test of the R language “limma” software package to screen differentially expressed genes. A total of 16 metabolic genes were screened out. Related data were analyzed by the “limma” and “igraph” R package. Twenty-three metabolic lncRNAs were identified. Then we analyzed metabolic lncRNAs related to survival using the “survival” R package. Samples were screened according to p < 0.05.

### Consensus clustering analysis

“Consensus Clusterplus” R package was used to investigate the expression characteristics of metabolic lncRNAs in GC and to cluster the patients into different groups. After that, the OS of GC patients in different groups was analyzed by “survival” R package. The association of expression pattern of metabolic lncRNAs and clinicopathologic features in different groups were visualized using “pheatmap” R package. Fisher test was performed to compare the distribution of each clinicopathological character.

### Immune infiltration analyses

“CIBERSORT” and “ESTIMATE” R packages were used to detect tumor-infiltrating immune cells and to compare the level of microenvironment scores among three clusters. The ESTIMATE algorithm based on single-sample GSEA was applied to infer the levels of immune cell responses and estimate the tumor purity in tumor samples among three clusters (16). The following three scores were derived from this algorithm: (1) StromalScore (the presence of matrix in tumor tissue); (2) EstimateScore (the inference of tumor purity); and (3) ImmuneScore (the infiltration of immune cells in tumor tissue). The immune cellular distributions of each GC sample were displayed by “barplot” package. The differential proportions of 22 immune cells among three clusters were visualized by the boxplot package in the R software. Immune cellular components and composition analysis was conducted using CIBERSORT (17).

### Procedure of the metabolic lncRNA model

Kaplan–Meier and univariate Cox regression analyses were conducted using the “survival” R package. Only significant genes (p<0.05) in both Kaplan–Meier and Cox analyses were considered potential prognostic metabolic genes. The LASSO (Least absolute shrinkage and selection operator) analysis with twelve cross-validations was conducted by applying the “glmnet” R package, with the best penalty parameter lambda. A prognostic gene list with coefficients was calculated by the LASSO model with the optimal lambda value. Then, each patient’s risk score was obtained from the gene expression levels and corresponding coefficients. We developed a metabolic lncRNA prognostic signature for the GC patients involving seven metabolic lncRNAs. Risk score = ∑_(i=n)^n〖Coefi*Xi〗 (where Coefi is the coefficient of each selected gene, Xi is the expression value). Patients with gastric cancer were randomly assigned to the training group and the testing group in a 1:1 ratio. Patients were divided into low- and high-risk groups by the cutoff value of the median value of risk scores. The survival difference between the two groups was conducted by Kaplan–Meier analysis. The prognostic ability of the gene signature was further assessed using Cox and receiver operating characteristic (ROC) analyses.

### Tumor mutation burden and TIDE analysis

Tumor mutation burden (TMB) analysis was adopted to analyze the mutational burden of tumors in the high- and low-risk groups. Combining the risk score and mutation compound, we divided GC patients into four groups and compared the survival between them. TIDE (tumor immune dysfunction and exclusion) scores were calculated by the website (http://tide.dfci.harvard.edu/) to assess whether patients could benefit from immunotherapy. Drug Sensitivity Analysis was conducted using the”pRRophetic” R package.

### Statistical analysis

Statistical analysis was performed with “Bioconductor” R packages. The prognostic ability of the derived prognostic signatures for GC in comparison to other clinicopathological characteristics was evaluated using ROC curve analysis [18]. The independent prognostic value of the risk scores for OS was evaluated using univariate and multivariate Cox proportional hazard regression analyses. Survival analysis of GC patients was conducted using the Kaplan–Meier method. A two-tailed p< 0.05 was considered to be statistically significant.

## Results

### Identification of metabolic lncRNAs

**Figure 1:**
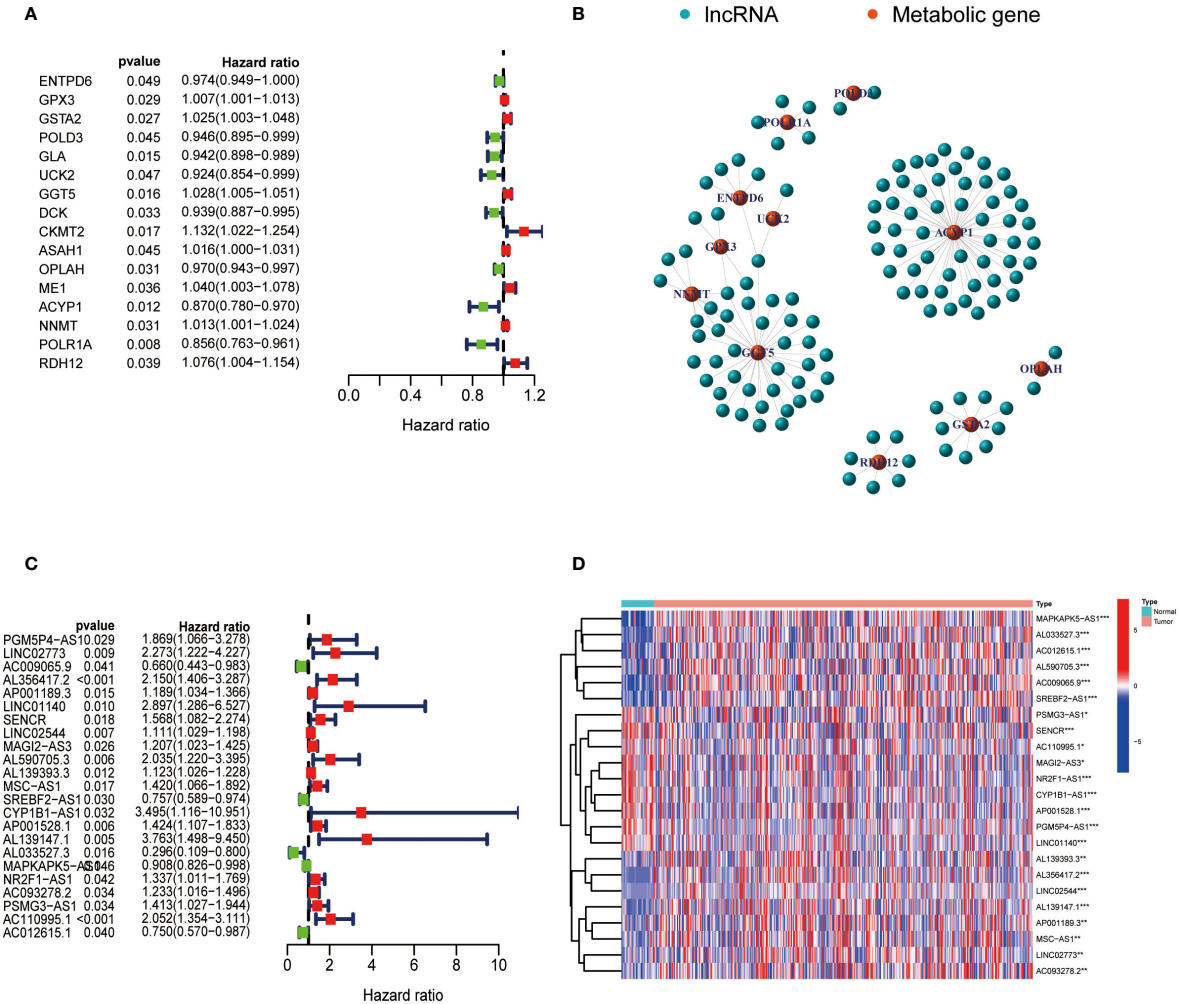
Identification of metabolic lncRNAs. **(A)** Metabolism-related genes significantly associated with the prognosis of GC patients. **(B)** Interaction network diagram for relationship between metabolism-related genes and their relationship with lncRNAs. **(C)** Forest plot of lncRNAs expression by one-way Cox analysis, where red represented high risk lncRNAs and green represented low risk lncRNAs. **(D)** Heatmap of metabolic lncRNAs expression in normal and tumor samples. Red represented upregulated expression, and blue represented downregulated expression. *p<0.05,**p < 0.01 and ***p < 0.001.

The expression data of metabolism-related genes were extracted from the transcriptome data of TCGA. **Figure 1A** showed metabolism-related genes which were significantly associated with the prognosis of GC patients. The correlation between the expression of lncRNAs and metabolism-related genes was analyzed by co-expression analysis. Network diagrams were generated to visually display this correlation (**Figure 1B**). The forest map showed the results of the cox-regression analysis, and lncRNAs were considered to be prognostic indicators when the p value was less than 0.05. Twenty-three lncRNAs were significantly correlated with the survival of GC and were selected as metabolic lncRNAs (**Figure 1C**). The expressions of these metabolic prognostic lncRNAs in tumor and normal tissues were analyzed (**Figure 1D**). As the results shown, the expression of all twenty-three metabolic lncRNAs showed significant difference in normal versus cancer tissue of GC.

### Construction of metabolic lncRNA patterns

**Figure 2:**
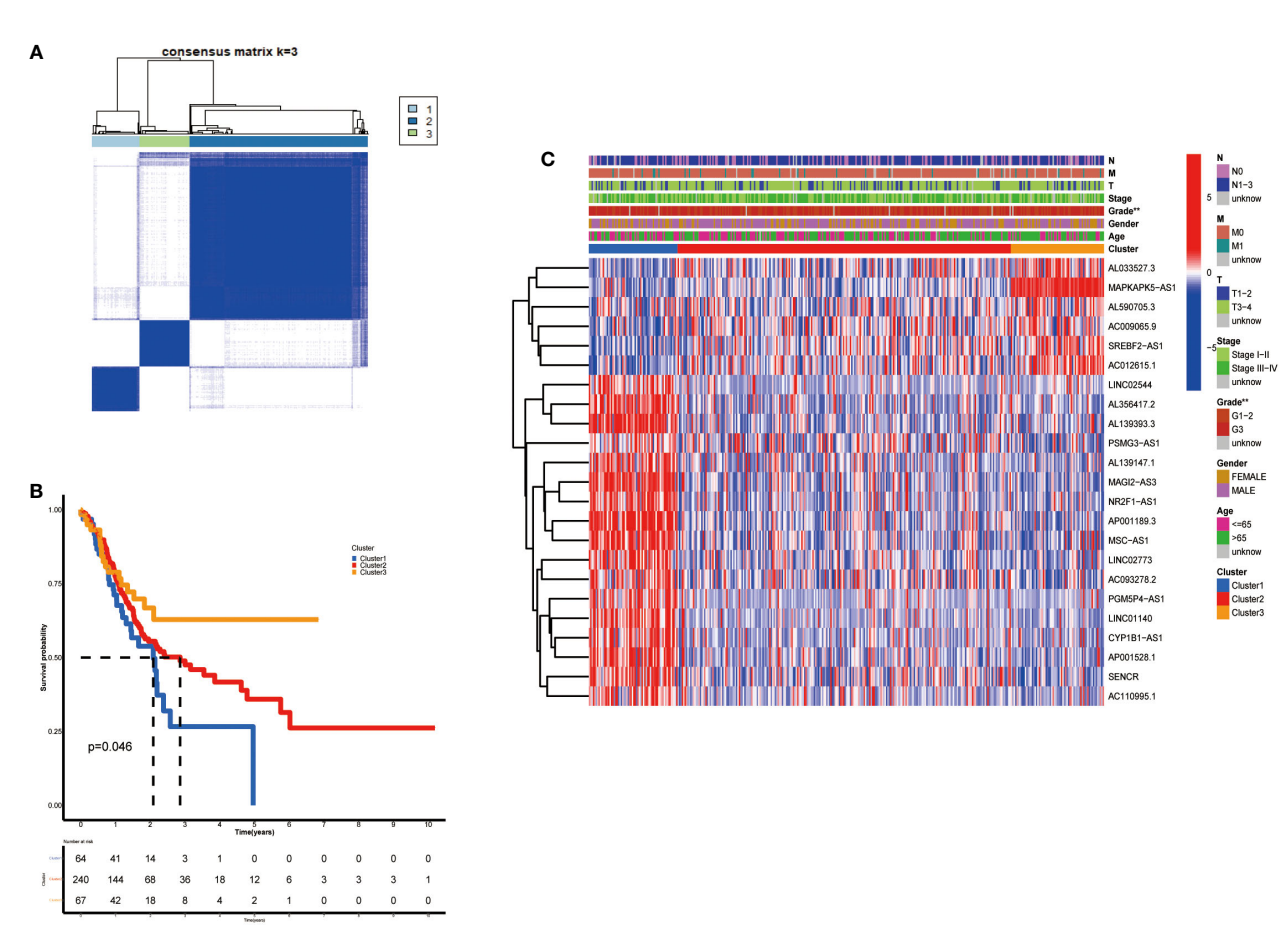
Construction of metabolic lncRNAs patterns. **(A)** Consensus clustering matrix for k = 3. **(B)** Kaplan–Meier analysis of patients in three metabolic lncRNAs patterns. **(C)** The clinicopathological differences among cluster 1, cluster 2 and cluster 3. **p < 0.01.

According to the analysis performed using the Consenses cluster Plus R package, metabolic lncRNAs in GC were divided into different clusters. When the consensus matrix k value was equal to 3, there was the least crossover among the GC samples. Therefore, we divided GC clusters into three types: cluster 1(n=64), cluster 2(n=240) and cluster 3(n=67) (**Figure 2A**; **Figure S1**). We ran a survival analysis according to the lncRNA patterns to evaluate the prognostic value of metabolic lncRNAs. As shown in the image, the OS of GC patients was significantly different among the three clusters (p=0.046). GC patients in cluster 1 suffered the worst OS (**Figure 2B**). The heatmap showed the expression of prognosis-related metabolic lncRNAs and their correlation with clinicopathological parameters in different clusters. No significant differences were detected among other clinical characteristics except for the grade of GC (**Figure 2C**).

### Immune cell infiltration of three clusters in GC

**Figure 3:**
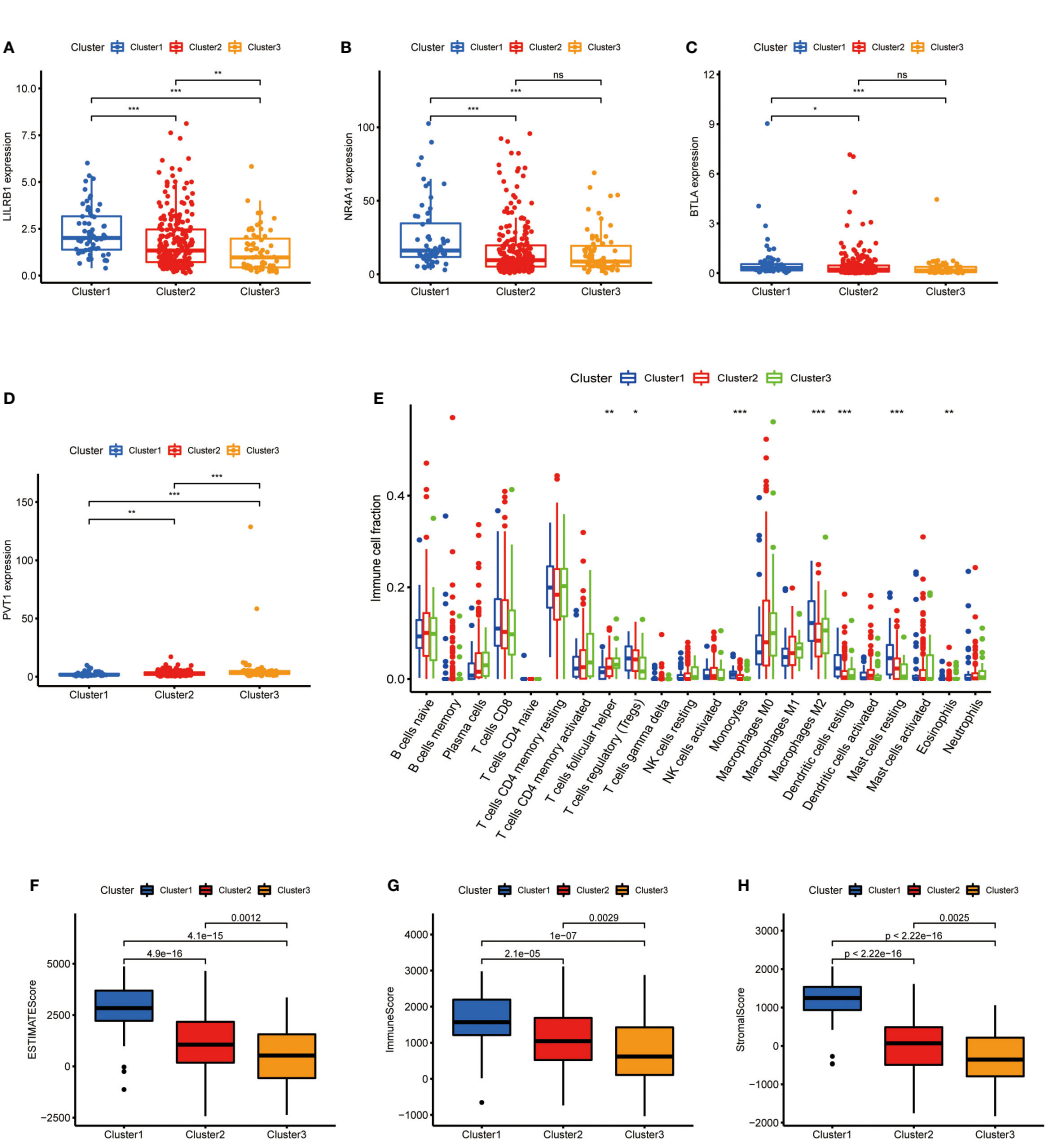
Immune cells infiltration of three clusters in GC. **(A–D)** Immune genes (LILRB1, NR4A1, BTLA) or oncogene (PVT1) expression of three clusters in GC. **(E)** Differences levels of infiltration of the 22 immune cells in three metabolic lncRNAs patterns. **(F–H)** The comparsion of ESTIMATEScore, ImmuneScore, and StromalScore in cluster 1, cluster 2 and cluster 3. *p<0.05,**p < 0.01 and ***p < 0.001.

After clarifying the predictive potential of metabolic lncRNAs clusters, we further explored the difference in immune infiltration among the patterns. We examined the expression of immune-related genes or oncogenes in three clusters, as shown in the **Figures 3A–D**, the expression of leukocyte immunoglobulin like receptor B1 (LILRB1), B and T lymphocyte associated (BTLA), homo sapiens nuclear receptor subfamily 4 (NR4A1) and plasmacytoma variant translocation 1 (PVT1) was varied among three clusters obviously. The CIBERSORT algorithm was used to estimate the fraction of 22 immune cell types in three clusters. The differentiation ratio of tumor immune cells in each cluster was shown by the boxplot diagram (**Figure 3E**). The distribution of immune cells such as monocytes, macrophages, mast cells and dendritic cells resting were distinctive (p<0.001). Moreover, the ESTIMATEScore, ImmuneScore and StromalScore decreased successively in the three groups, where cluster 3 got the lowest score (**Figures 3F–H**). Collectively, the above findings indicated that the cluster pattern based on metabolic lncRNAs is reliable to distinguish the prognosis and immune status of GC.

### Establishment of risk score model

**Figure 4:**
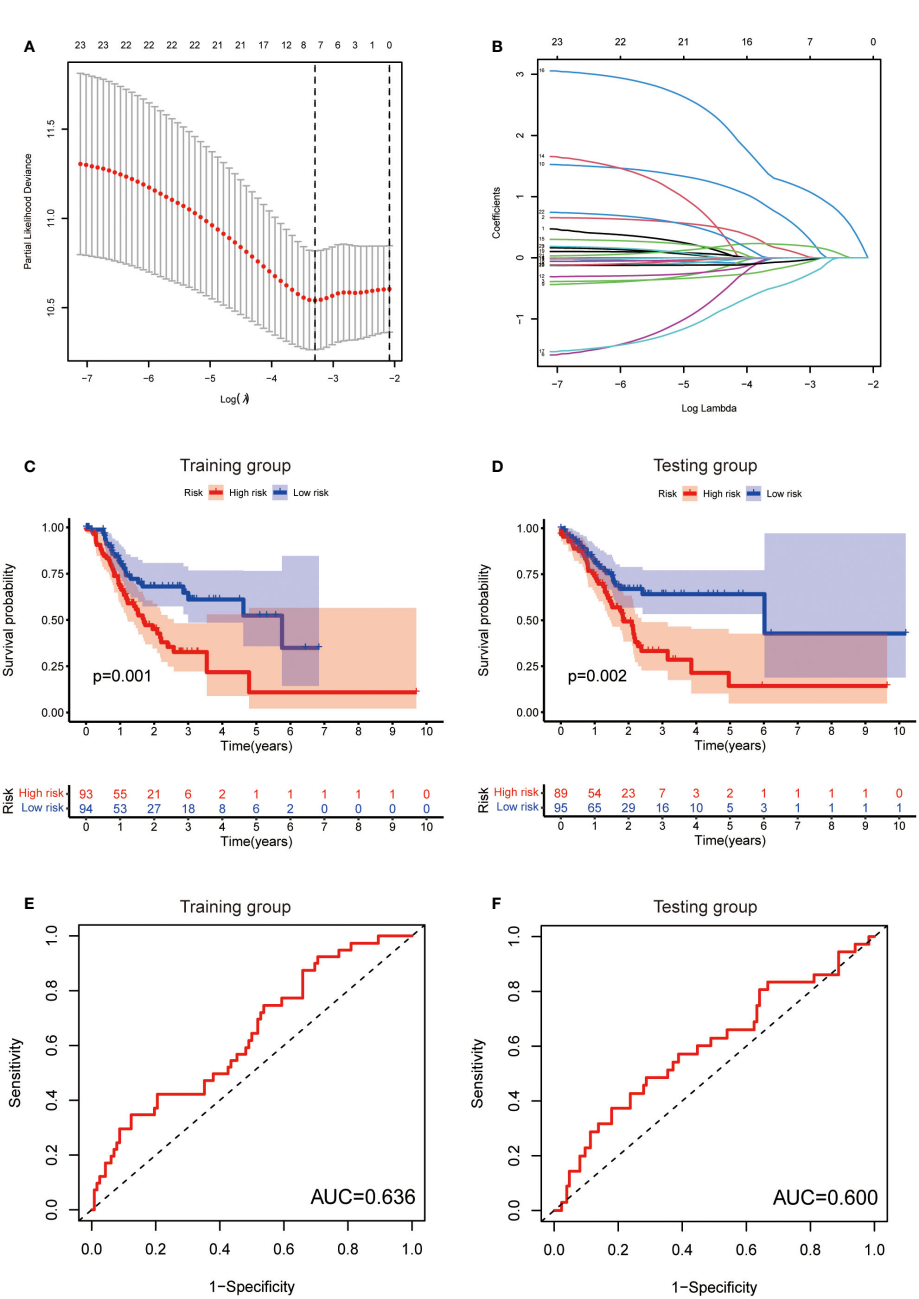
Establishment of risk score model. **(A)** The distribution of lambda and the best options in Lasso analysis **(B)** The weight of each candidate gene in the model. **(C, D)** Kaplan-Meier survival curves of the OS of patients between the high- and low-risk groups in the training **(C)** and testing **(D)** set. **(E, F)** The ROC curves of the risk score model in training **(E)** and testing **(F)** group.

**Figure 5:**
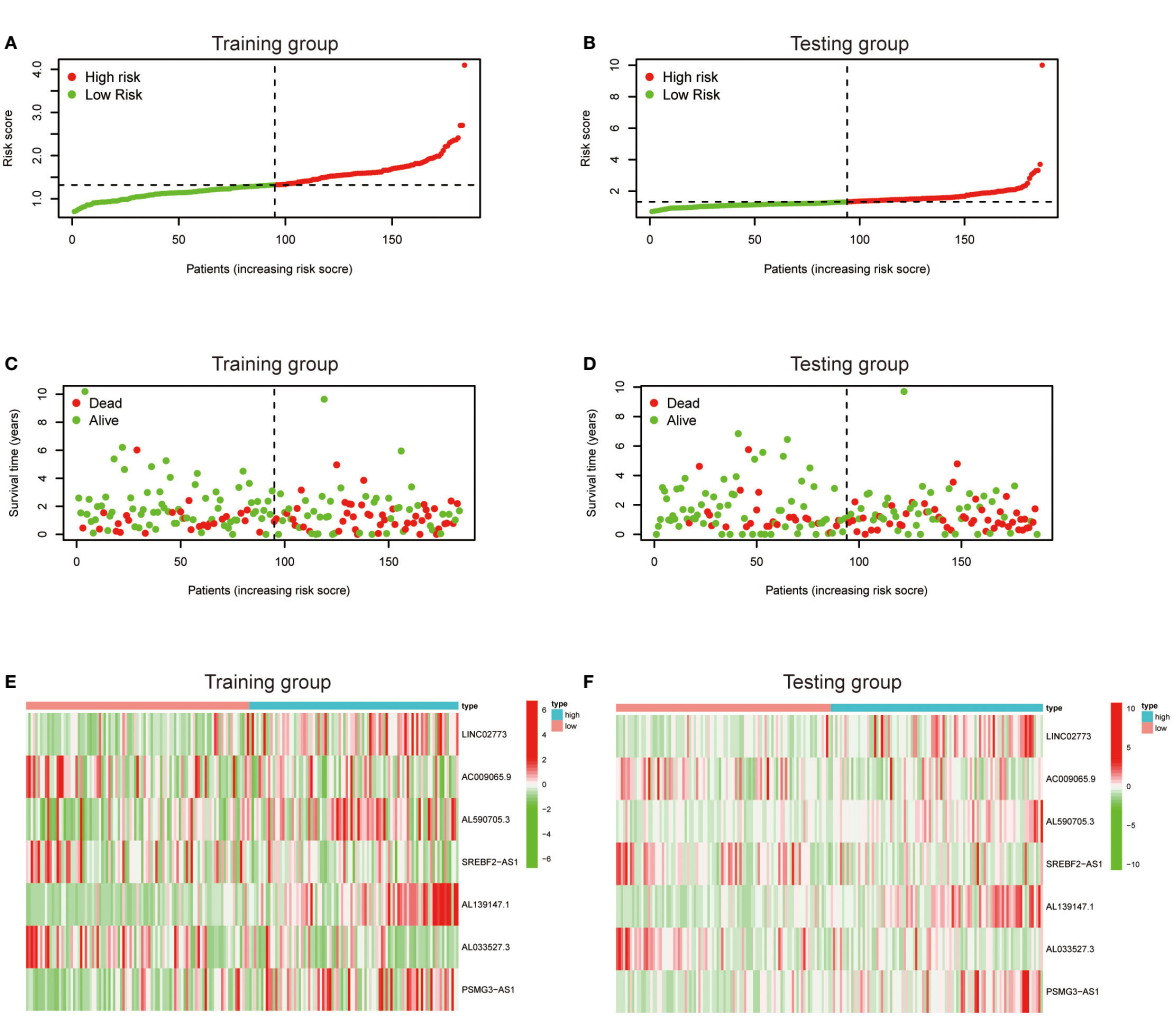
Prognostic value of the risk score model. **(A, B)** Patterns of survival status and survival time in the training and testing group. **(C, D)** Distribution of metabolic lncRNAs risk score model in the high- and low-risk groups plotted in training and testing set. **(E, F)** Heatmap showed the expression standards of the seven prognostic lncRNAs of training and testing set.

To further exploit the predictive value of metabolic lncRNAs, Lasso Cox regression was used to construct the risk score model based on the TCGA database (**Figures 4A, B**). Seven genes were selected and adopted to build the risk score model. Risk score=0.105*LINC02773 – 0.134* AC009065.9 + 0.579*AL590705.3 – 0.057* SREBF2-AS1 +1.207* AL139147.1 – 0.397* AL033527.3 + 0.211* PSMG3-AS1. To verify the predictive value of the model, we divided the samples of the training (n=187) and testing group (n=184) into high-risk group and low-risk group according to the median value of the risk scores. The OS of high-risk and low-risk patients in the two groups differed significantly (**Figures 4C, D**). Patients in the high-risk group suffered a worse OS (p<0.05). The AUC value illustrated that the risk model has acceptable performance in predicting the prognosis of the two groups of patients (**Figures 4E, F**). **Figures 5A–D** represented the survival status of gastric cancer patients in training and testing groups respectively. The survival status and risk score distribution in the TCGA training and testing datasets indicated that the proportion of patients who died was considerably greater in those with high scores as opposed to those with low scores. Heatmap displayed that the expression level of the 4 genes increased as the score increased (**Figures 5E, F**). Our findings indicated that the metabolic lncRNA risk model can serve as a reliable indicator to predict the prognosis of GC patients.

### Metabolic lncRNAs model was an independent prognostic factor

**Figure 6:**
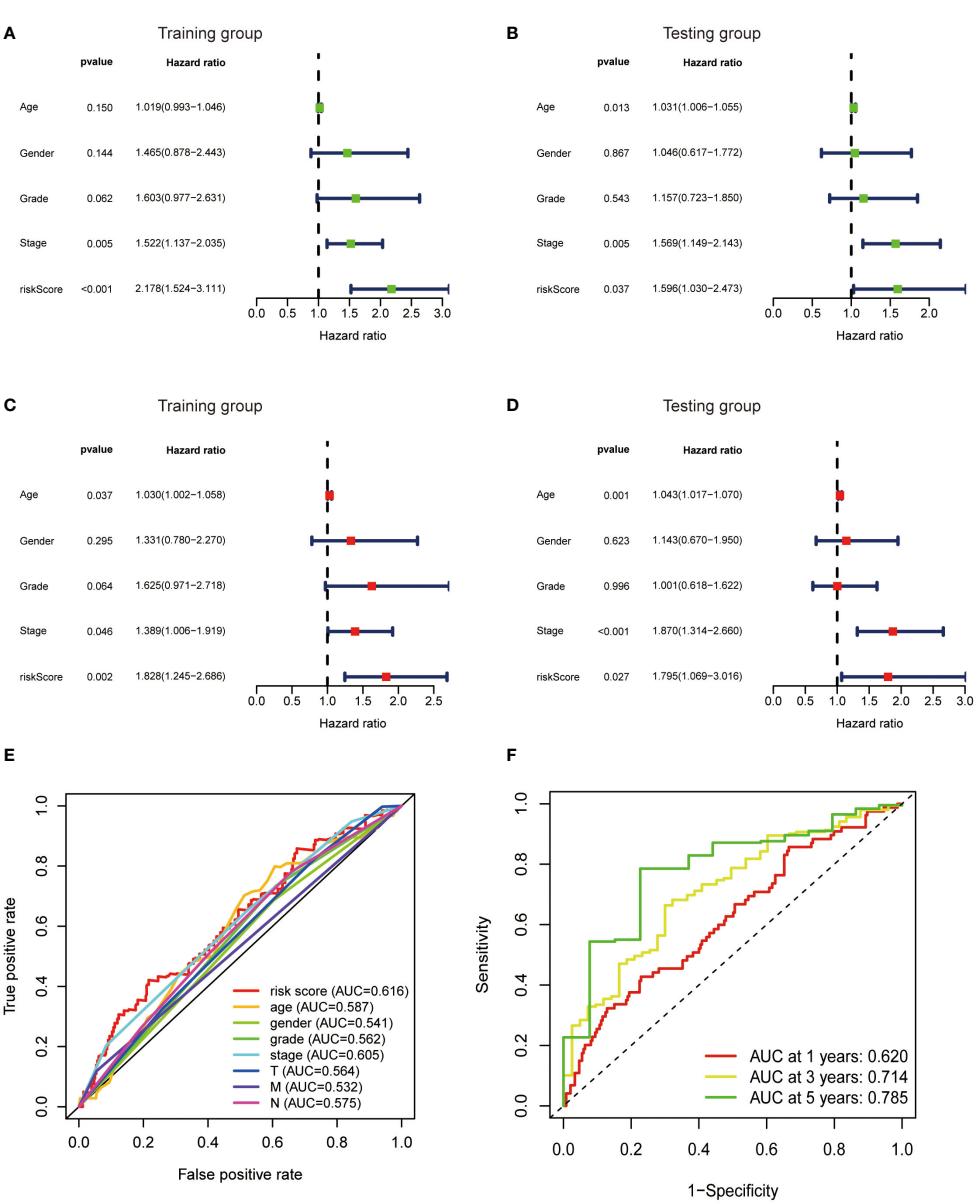
Metabolic lncRNAs risk score model was an independent prognostic factor. Univariate **(A, B)** and multivariate **(C, D)** Cox regression analysis of the association between clinicopathological features (including risk score) and OS of patients in the training and testing group. **(E)** The ROC curves of the risk score model and clinicopathological parameters **(F)** The ROC curves of the risk score model at 1-,3-,5-years.

To further verify the predictive value of the risk model, univariate and multivariate regression analysis including risk model and clinicopathological parameters such as age, gender, grade and stage were conducted. Both univariate and multivariate analysis showed that the metabolic lncRNA risk model significantly correlated with OS, illustrating that the risk model was an independent prognostic factor for GC patients (**Figures 6A–D**). It was found that the diagnostic value of risk model is better than clinicopathological features, including the TNM staging (**Figure 6E**). Moreover, the AUC value increased with the year (**Figure 6F**).

### Validation of the prognostic risk model in clinicopathological features of GC

**Figure 7:**
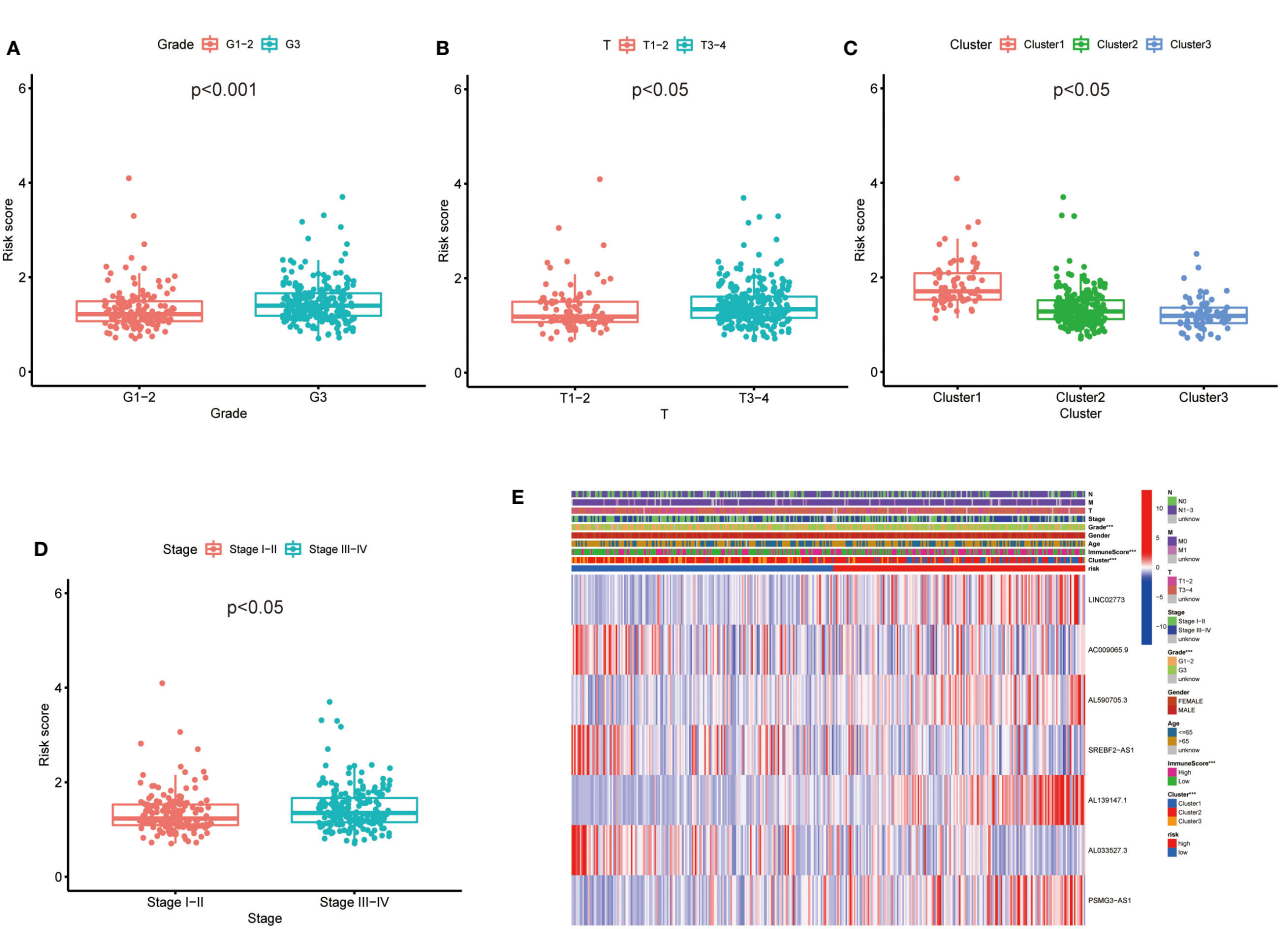
The prognostic risk model was applied to clinical features and immune characteristics of GC patients **(A–D)** Boxplot of relationship analysis of risk score and clinical features. **(E)** The clinicopathological differences between the high- and low-risk groups. ***p < 0.001.

**Figure 8:**
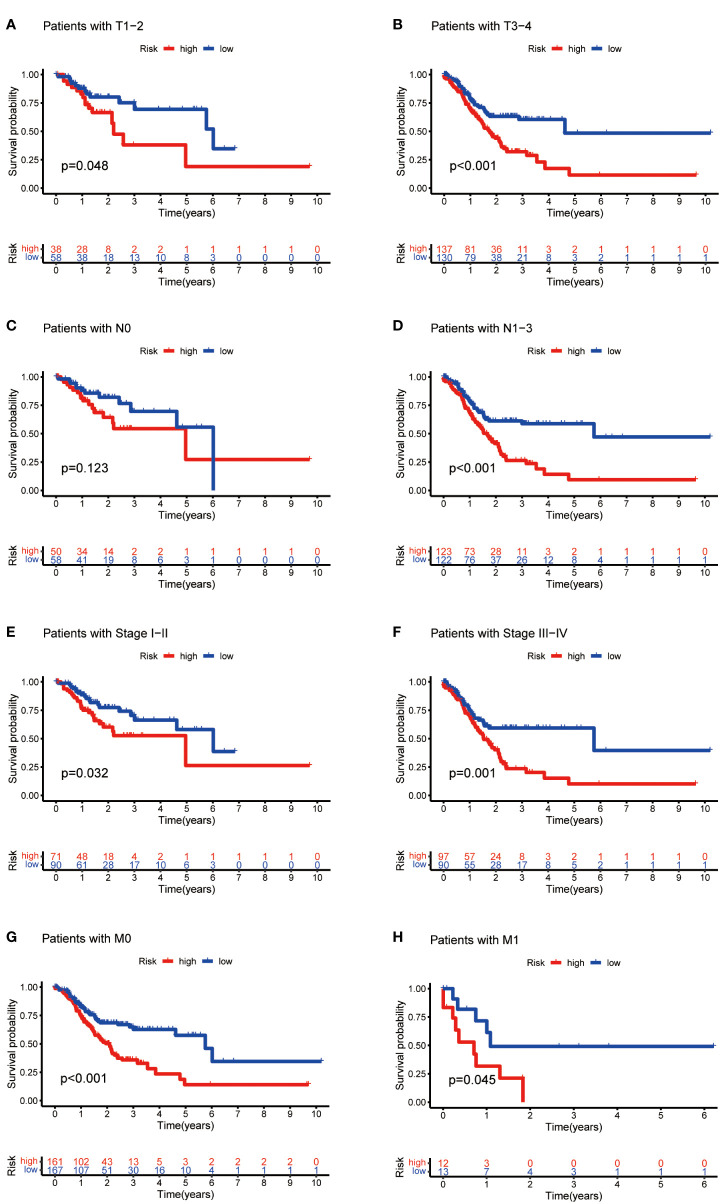
Kaplan-Meier curves of OS differences stratified by tumor invasion depth (T), lymph node matestasis (N), distinal metastasis (M) and TNM staging between the high- and low-risk groups.

Then, we evaluated the correlation between the risk score and clinicopathological features of GC patients. The grade, tumor invasion depth, cluster pattern and TNM staging showed significant differences in the high- and low-risk groups (**Figures 7A–D**). The expression of the seven selected metabolic lncRNAs were varied, as shown in the heatmap, LINC02773, AL590705.3, AL139147.1 and PSMG3-AS1 were highly expressed, while AC009065.9, SREBF2-AS1 and AL033527.3 were expressed lowly in high-risk group(**Figure 7E**). Next, stratification analysis was performed to verify whether the risk score can maintain its prediction ability in each subgroup. The results showed the lncRNA risk score could further distinguish the survival difference among GC patients with same age, gender, grade, tumor invasion depth (T), lymph node metastasis (N), distal metastasis (M) and TNM staging (**Figure 8**; **Figure S2**).

### Metabolic lncRNAs risk model reflected the immune microenvironment of GC

**Figure 9:**
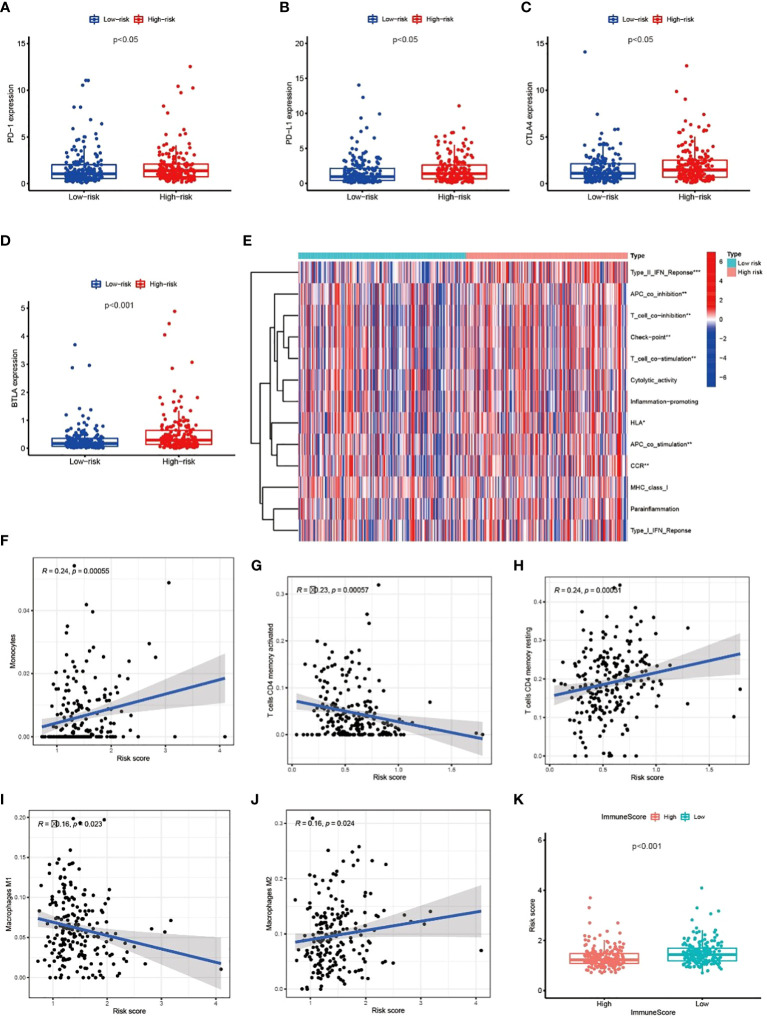
Metabolic lncRNAs risk model reflected the immune microenvironment **(A–D)** The relationship between immune checkpoint molecules and risk scores. **(E)** Heatmap of differences in immune function between high- and low-risk group. **(F–J)** The relationship between immune cell types and risk scores. **(K)** Boxplot of relationship analysis of risk score and ImmuneScore. *p < 0.05,**p < 0.01, ***p < 0.001.

We explored the correlation between immune genes or immune cell types and risk scores in GC patients. The results revealed that some key immune checkpoint molecules were significantly overexpressed in the high-risk group, such as CTLA4 (Cytotoxic T-lymphocyte associated protein 4), PD-1 (Programmed cell death 1) and corresponding ligand PD-L1(Programmed cell death 1 ligand 1), indicating that high-risk GC tends to be immunosuppressive (**Figures 9A–D**; **Figure S3**). Consistently, heatmap showed that genes involved in APC (Antigen-presenting cells) inhibition, T cell inhibition and checkpoint were significantly elevated in high-risk patients (**Figure 9E**). The antitumor immune cells (such as activated CD4+ T cell and macrophages M1 cell) decreased in the high-risk group, but tumor tolerant immune cells (such as resting CD4+ T cell and macrophages M2 cell) increased in the tumor microenvironment (p<0.05, **Figures 9F–J**; **Figure S4**). Consequently, the ImmuneScore decreased significantly in high-risk group (**Figure 9K**). These results suggested that there is an active interaction between abnormal tumor metabolism and the immune microenvironment. The metabolic lncRNA model can reflect the immune microenvironment of GC.

### Metabolic lncRNAs risk model predicted immunotherapy efficacy of GC

**Figure 10:**
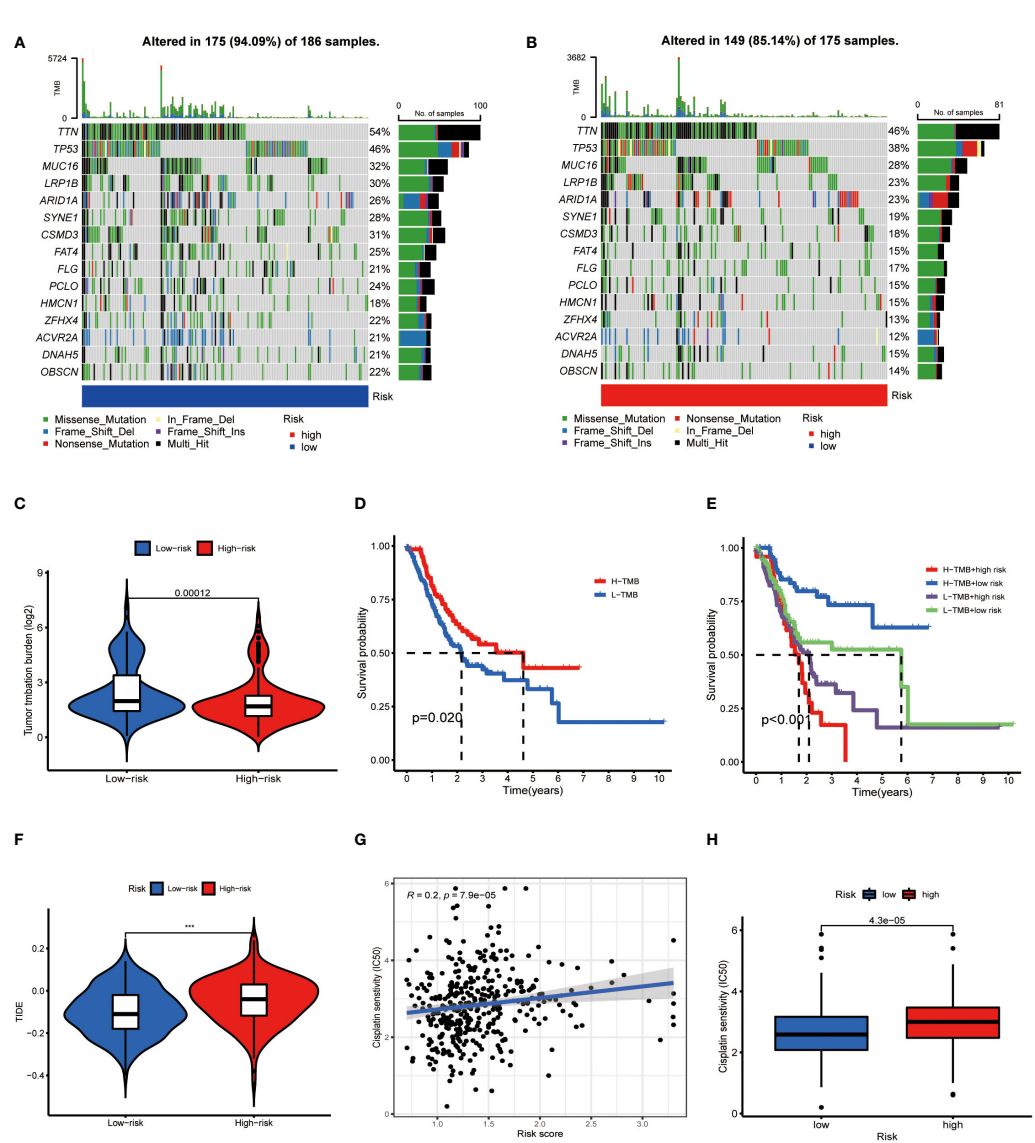
Metabolic lncRNAs risk model predicted immunotherapy efficacy. **(A, B)** Waterfall diagram of high- low-risk GC patients. **(C)** Tumor mutation burden analysis to compare the TMB of GC patients in two groups. **(D,E)** Kaplan-Meier analysis to compare the OS of GC patients in different groups. **(F)** Violin diagram showed the TIDE values of GC patients in two groups. **(G,H)** Sensitivity of patients with different risk scores to cisplatin treatment.

On the other side of the coin, whether tumor cells were easily recognized by the immune system was unknown. To figure this out, we focused on the TMB of GC. The waterfall chart showed the number of mutations in low- and high-risk GC (**Figures 10A, B**), mutation burden of high-risk GC was significantly lower than that of low-risk GC (**Figure 10C**). Survival analysis found that patients with high mutation burden have better OS in GC (**Figure 10D**), in particular, the OS of low-risk patients with high TMB was extremely longer than other types of patients (**Figure 10E**). TIDE value of low-risk GC patients was significantly lower than high-risk patients (**Figure 10F**). Moreover, drug sensitivity analysis found that patients with low-risk GC were more sensitive to cisplatin, which is a common drug in GC chemotherapy (**Figures 10G, H**). Collectively, low-risk GC patients were accompanied by active immune status and high tumor mutations, which illustrated these patients are more likely to benefit from immunotherapy.

## Discussion

The length of a patient’s survival time, in essence, reflects the level of malignancy of tumors. At present, TNM staging is the main method to judge the prognosis of GC patients. However, TNM staging is a representation of tumor progression but cannot accurately indicate the intrinsic properties of tumors, so cases of patients in the same TNM stage but with significantly different survival time is common. Accurate judgment of patients’ feedback on treatment and prediction of patients’ survival time are vital for the formulation of individualized treatment strategies. Therefore, it is necessary to find a more accurate method to predict the prognosis of GC patients.

N6-methyladenosine (m6A) is the most abundant RNA modification in eukaryotic cells (15). Over m6A modification of certain genes could lead to alterations of mRNA behavior and expression, resulting in the acceleration of tumor development, whereas lacking of m6A modification on other genes may also lead to tumor progression (16). Some researchers who explored the correlation between m6A related lncRNAs and immune infiltration, reported the value of these lncRNAs in predicting tumor prognosis. Zhou et al. reported that m6A-related lncRNAs could predict outcomes of head and neck squamous cell carcinoma and could provide new therapeutic targets for these patients (17). A prognostic model based on m6A-associated lncRNAs is a predictor of overall survival, which can also be used as a predictor of immunotherapy effectiveness and need (18). Unfortunately, although abnormal metabolism is irreplaceable in tumorigenesis and progression, the possibility of metabolic lncRNAs risk model in predicting the prognosis of malignant tumor is unknown.

In this study, we found that the predictive value of metabolic lncRNA risk model is higher than TNM staging (**Figure 6E**). This risk score was an independent predictor and was able to compensate for the inaccuracy of TNM staging. As shown in **Figure 8**, the metabolic lncRNA risk score could further differentiate the survival time of patients in early(stage I-II) or advanced (stage III-IV) stages, which can avoid unrealistic optimism about the prognosis of GC patients with early stage and excessive pessimism with advanced stages. We noticed this to be true even in advanced stage patients who already suffered distant metastasis (M1), some of whom were worthy of and would benefit from active treatment.

Immunotherapy is a breakthrough in cancer therapy in recent years. The latest research reports that immunotherapy brings encouraging efficacy in the treatment of advanced GC (19–21). However, the main dilemma for oncologists is the lack of reliable indicators to screen sensitive patients to immunotherapy. Understanding the immune status of different patients makes immunotherapy more reasonable and effective (22). Metabolic lncRNAs risk model revealed the immune environment of GC in two aspects. Firstly, the expression of negative regulatory receptors and corresponding ligands were increased in high-risk patients (**Figures 9A–E**), which inhibited the activation of T cells. Secondly, the number of anti-tumor immune cells decreased with the increase of risk score (**Figures 9F–J**). The above phenomena showed that high-risk GC goes hand-in-hand with immunosuppressive status. Nevertheless, immune status alone is not enough to predict the sensitivity of immunotherapy, because tumor cells can escape the specific immune recognition by T cells through down-regulating the expression of tumor specific antigen or related antigen (23). Tumor mutations may express more antigens recognized by immune cells to activate the immune system (24, 25), thus the guidelines recommend high TMB as an indication of immunotherapy in GC.

Obviously, the ideal indicator should reflect both tumor immune status and TMB, but the commonly used CPS (Combined positive score)/TPS (Tumor proportion score) or dMMR (Different mismatch repair)/MSI-H (Microsatellite instability-high) does not meet this requirement. Our study found that the risk model based on metabolic lncRNAs not only reflects the immune state, but is also significantly correlates with TMB. For instance, GC patients in the low-risk group tend to be in a significantly active immune status (**Figure 9**) and carry more TMB (**Figures 10A–C**). We found that immune checkpoint inhibitors are more likely to benefit patients with this feature, which is consistent with the results of TIDE analysis (**Figure 10F**). Thus, the metabolic lncRNAs risk model has great potential to serve as an indicator for screening immunotherapy sensitive GC patients.

We previously conducted deep exploration around the lncRNA PVT1 and found that PVT1 can promote GC neovascularization by activating the STAT3 (Signal transducer and activator of transcription 3) pathway and is able to regulate the BCL2 (B-cell lymphoma 2) protein which leads to GC resistance to 5-Fluorouracil (5-FU) (26, 27). After classifying GC samples based on metabolic lncRNAs, we found that the expression of PVT1 was significantly different among differing patterns and risk score models. This reminded us that PVT1 may be associated with metabolic and immune status, but the specific mechanism of PVT1 deserves further exploration.

Despite the robust prognostic risk model of seven lncRNA established in this study, several limitations of our study remain. First, our results were obtained and validated using the TCGA dataset, more independent gastric cancer cohorts should be used to validate the risk model of seven metabolic lncRANs. Second, this study was a bioinformatic and retrospective study, further cell line and animal functional experiments were needed to reveal the intrinsic mechanisms of prognostic lncRNAs. Finally, we were not able to verify theirs specific biological functions and found the exact signaling pathways of metabolic lncRNAs.

In conclusion, our study used microarray data from 372 GC samples to screen out key genes related to metabolism, and built an independent prediction model based on metabolic lncRNAs. The present pilot study revealed that the metabolic lncRNAs model is significantly associated with the immune environment and TMB, and strongly suggests that the risk model is a reliable indicator to predict the efficacy of immunotherapy. We hope that our findings can contribute to a deeper understanding of the relationship between metabolism and immunity, help provide a new perspective in predicting GC prognosis, and help provide indications for immunotherapy of GC.

## Data availability statement

The datasets presented in this study can be found in online repositories. The names of the repository/repositories and accession number(s) can be found in the article/**Supplementary Material**.

## Author contributions

GH and YL: designed the study. PD and CH: analyzed, interpreted the data, and wrote original draft. PD, PL, SC and RP: wrote this manuscript. All authors contributed to the article and approved the submitted version.
