# Supplementary material for: The value of metabolic LncRNAs in predicting prognosis and immunotherapy efficacy of gastric cancer
Source: Front Oncol. 2023 Jan 4;12:1019909. doi: 10.3389/fonc.2022.1019909 (PMC9845566; doi:10.3389/fonc.2022.1019909)
Supplement: Supplementary file 2 [file DataSheet_2.docx]

TableS1. Metabolism-related genes significantly related to the prognosis of GC patients in TCGA dataset

| Gene | HR | HR.95L | HR.95H | pvalue |
| --- | --- | --- | --- | --- |
| ENTPD6 | 0.97 | 0.95 | 1.00 | 0.05 |
| GPX3 | 1.01 | 1.00 | 1.01 | 0.03 |
| GSTA2 | 1.03 | 1.00 | 1.05 | 0.03 |
| POLD3 | 0.95 | 0.90 | 1.00 | 0.05 |
| GLA | 0.94 | 0.90 | 0.99 | 0.02 |
| UCK2 | 0.92 | 0.85 | 1.00 | 0.05 |
| GGT5 | 1.03 | 1.01 | 1.05 | 0.02 |
| DCK | 0.94 | 0.89 | 1.00 | 0.03 |
| CKMT2 | 1.13 | 1.02 | 1.25 | 0.02 |
| ASAH1 | 1.02 | 1.00 | 1.03 | 0.04 |
| OPLAH | 0.97 | 0.94 | 1.00 | 0.03 |
| ME1 | 1.04 | 1.00 | 1.08 | 0.04 |
| ACYP1 | 0.87 | 0.78 | 0.97 | 0.01 |
| NNMT | 1.01 | 1.00 | 1.02 | 0.03 |
| POLR1A | 0.86 | 0.76 | 0.96 | 0.01 |
| RDH12 | 1.08 | 1.00 | 1.15 | 0.04 |

Table S2.The lncRNAs related to metabolism-related genes in gastric cancer.

| Metabolic genes | lncRNA | cor | pvalue |  | Metabolic genes | lncRNA | cor | pvalue |
| --- | --- | --- | --- | --- | --- | --- | --- | --- |
| ACYP1 | AC017083.1 | 0.44 | 7.54E-19 |  | **ACYP1** | AL139289.1 | 0.45 | 3.65E-20 |
| ACYP1 | AC005519.1 | 0.43 | 4.33E-18 |  | **GGT5** | AC016924.1 | 0.53 | 5.89E-28 |
| UCK2 | AL358115.1 | 0.43 | 2.90E-18 |  | **ACYP1** | AL136304.1 | 0.43 | 3.13E-18 |
| GGT5 | AC009318.2 | 0.52 | 1.11E-27 |  | **ACYP1** | AL355488.1 | 0.50 | 7.41E-25 |
| RDH12 | AC116407.1 | 0.47 | 3.34E-22 |  | **ACYP1** | AL445222.2 | 0.43 | 1.21E-18 |
| ACYP1 | AC012213.3 | 0.43 | 2.66E-18 |  | **ACYP1** | SNHG10 | 0.57 | 1.25E-33 |
| ACYP1 | STAM-AS1 | 0.40 | 4.36E-16 |  | **POLR1A** | SMG7-AS1 | 0.40 | 5.72E-16 |
| ACYP1 | AC116407.2 | 0.40 | 4.20E-16 |  | **GGT5** | AC026356.1 | 0.40 | 3.69E-16 |
| GGT5 | BNC2-AS1 | 0.42 | 9.72E-18 |  | **ACYP1** | SREBF2-AS1 | 0.40 | 3.48E-16 |
| ACYP1 | AL451050.2 | 0.44 | 6.63E-19 |  | **GGT5** | AC138207.5 | 0.42 | 9.42E-18 |
| ENTPD6 | AL035252.4 | 0.47 | 5.50E-22 |  | **RDH12** | AC008268.1 | 0.59 | 2.59E-36 |
| RDH12 | C9orf147 | 0.53 | 2.17E-28 |  | **GGT5** | AL109741.1 | 0.50 | 6.63E-25 |
| GGT5 | AC008808.2 | 0.44 | 3.78E-19 |  | **GSTA2** | AP005057.1 | 0.42 | 2.93E-17 |
| GGT5 | AC107959.1 | 0.46 | 4.53E-21 |  | **GGT5** | CYP1B1-AS1 | 0.47 | 3.68E-22 |
| ACYP1 | AC034229.4 | 0.43 | 2.17E-18 |  | **GGT5** | AP001528.1 | 0.53 | 5.22E-28 |
| RDH12 | LINC02057 | 0.51 | 1.31E-26 |  | **ACYP1** | AL158063.1 | 0.41 | 2.99E-16 |
| ACYP1 | LINC00115 | 0.40 | 6.88E-16 |  | **NNMT** | LINC01235 | 0.44 | 1.57E-19 |
| ACYP1 | SNHG1 | 0.44 | 2.65E-19 |  | **GPX3** | AL133415.1 | 0.45 | 5.85E-20 |
| GPX3 | AC002546.1 | 0.50 | 2.18E-25 |  | **GGT5** | AL133415.1 | 0.63 | 1.62E-43 |
| GGT5 | PGM5P4-AS1 | 0.45 | 1.56E-20 |  | **NNMT** | AL133415.1 | 0.40 | 6.87E-16 |
| GSTA2 | LINC00242 | 0.40 | 3.24E-16 |  | **ACYP1** | LINC02803 | 0.41 | 1.67E-16 |
| GGT5 | AC009318.3 | 0.46 | 3.25E-21 |  | **NNMT** | AL139147.1 | 0.40 | 5.32E-16 |
| ACYP1 | WASHC5-AS1 | 0.47 | 5.30E-22 |  | **GGT5** | AC080038.2 | 0.50 | 6.06E-25 |
| GGT5 | LINC02773 | 0.52 | 6.15E-28 |  | **NNMT** | AC080038.2 | 0.43 | 2.27E-18 |
| GSTA2 | AP001065.4 | 0.44 | 9.04E-19 |  | **GGT5** | LINC01537 | 0.45 | 5.00E-20 |
| ACYP1 | AC009065.9 | 0.42 | 3.07E-17 |  | **GGT5** | AL445426.1 | 0.54 | 4.91E-30 |
| ACYP1 | SCAT2 | 0.48 | 1.34E-22 |  | **ACYP1** | SNHG30 | 0.40 | 6.96E-16 |
| ACYP1 | AL355802.3 | 0.41 | 8.46E-17 |  | **GGT5** | MIR497HG | 0.48 | 1.56E-22 |
| GGT5 | MIR99AHG | 0.43 | 1.34E-18 |  | **ACYP1** | AC021851.1 | 0.42 | 3.01E-17 |
| ENTPD6 | MIR100HG | -0.41 | 1.71E-16 |  | **GGT5** | AC120049.1 | 0.47 | 3.80E-22 |
| ACYP1 | AZIN1-AS1 | 0.45 | 1.66E-20 |  | **ACYP1** | AP001160.1 | 0.44 | 1.94E-19 |
| GGT5 | LINC02593 | 0.44 | 5.35E-19 |  | **ACYP1** | AL033527.3 | 0.43 | 1.62E-18 |
| POLR1A | OGFRP1 | 0.46 | 2.01E-21 |  | **GSTA2** | AL355096.1 | 0.44 | 6.81E-19 |
| GGT5 | AL356417.2 | 0.47 | 4.29E-22 |  | **ACYP1** | MAPKAPK5-AS1 | 0.46 | 6.81E-21 |
| NNMT | AL356417.2 | 0.59 | 1.51E-36 |  | **GGT5** | GAS1RR | 0.47 | 5.13E-22 |
| ENTPD6 | AC124067.4 | 0.42 | 1.11E-17 |  | **ENTPD6** | NR2F1-AS1 | -0.42 | 2.35E-17 |
| GGT5 | AP001189.3 | 0.63 | 4.27E-42 |  | **GGT5** | AC012636.1 | 0.45 | 8.94E-20 |
| GPX3 | LINC01140 | 0.43 | 3.21E-18 |  | **POLD3** | AC079466.1 | 0.43 | 4.85E-18 |
| GGT5 | LINC01140 | 0.49 | 3.24E-24 |  | **ACYP1** | LINC01355 | 0.45 | 3.34E-20 |
| OPLAH | AC233992.3 | 0.42 | 2.05E-17 |  | **GGT5** | AC093278.2 | 0.42 | 4.52E-17 |
| GGT5 | AL359853.1 | 0.57 | 1.71E-33 |  | **ACYP1** | AC090517.2 | 0.42 | 1.92E-17 |
| ACYP1 | AL049780.1 | 0.41 | 2.39E-16 |  | **NNMT** | Z97200.1 | 0.44 | 6.85E-19 |
| RDH12 | AC103563.7 | 0.49 | 2.76E-24 |  | **ACYP1** | AL139089.1 | 0.58 | 1.42E-34 |
| ACYP1 | MHENCR | 0.40 | 5.98E-16 |  | **ACYP1** | AL158196.1 | 0.40 | 4.69E-16 |
| ACYP1 | AC008761.2 | 0.42 | 1.31E-17 |  | **POLR1A** | AC099850.4 | 0.50 | 1.08E-24 |
| ACYP1 | RAB30-DT | 0.41 | 2.19E-16 |  | **ACYP1** | RNF139-AS1 | 0.40 | 3.79E-16 |
| GGT5 | SENCR | 0.67 | 3.82E-50 |  | **ACYP1** | SNHG12 | 0.45 | 2.07E-20 |
| NNMT | SENCR | 0.45 | 4.67E-20 |  | **GGT5** | PSMG3-AS1 | 0.41 | 1.13E-16 |
| GSTA2 | AC093583.1 | 0.41 | 1.90E-16 |  | **ACYP1** | DDX11-AS1 | 0.43 | 2.57E-18 |
| GPX3 | LINC02544 | 0.41 | 1.63E-16 |  | **ACYP1** | AC093157.1 | 0.41 | 1.11E-16 |
| ENTPD6 | MAGI2-AS3 | -0.43 | 5.49E-18 |  | **GGT5** | AC110995.1 | 0.42 | 2.17E-17 |
| UCK2 | MAGI2-AS3 | -0.44 | 1.53E-19 |  | **ACYP1** | AL109811.1 | 0.42 | 1.62E-17 |
| GGT5 | MAGI2-AS3 | 0.48 | 1.08E-22 |  | **POLD3** | AP001372.2 | 0.59 | 1.74E-36 |
| POLR1A | AC073046.1 | 0.46 | 5.34E-21 |  | **GGT5** | HHIP-AS1 | 0.43 | 2.15E-18 |
| ACYP1 | AL590705.3 | 0.43 | 5.21E-18 |  | **ACYP1** | AC024060.2 | 0.47 | 4.03E-22 |
| ACYP1 | AL390728.6 | 0.42 | 3.06E-17 |  | **GSTA2** | ADORA2A-AS1 | 0.60 | 8.36E-38 |
| ACYP1 | AC253536.6 | 0.40 | 7.15E-16 |  | **ACYP1** | DLEU1 | 0.44 | 2.99E-19 |
| GSTA2 | AC008738.2 | 0.40 | 3.30E-16 |  | **GGT5** | AC125807.2 | 0.43 | 1.61E-18 |
| ACYP1 | AL662797.2 | 0.40 | 4.27E-16 |  | **ACYP1** | AC012615.1 | 0.49 | 4.48E-24 |
| GGT5 | AL139393.3 | 0.43 | 6.55E-18 |  | **ACYP1** | AC007038.1 | 0.51 | 3.46E-26 |
| NNMT | AL139393.3 | 0.41 | 1.07E-16 |  | **RDH12** | PRDM16-DT | 0.40 | 5.16E-16 |
| ACYP1 | RUSC1-AS1 | 0.40 | 6.12E-16 |  | **OPLAH** | AC084125.2 | 0.43 | 6.70E-18 |
| RDH12 | NCOA7-AS1 | 0.52 | 5.27E-27 |  | **GSTA2** | AC000068.1 | 0.50 | 5.20E-25 |
| POLR1A | AC012073.1 | 0.46 | 2.22E-21 |  | **GGT5** | AC011899.2 | 0.44 | 3.63E-19 |
| ACYP1 | INTS6-AS1 | 0.43 | 1.07E-18 |  | **ACYP1** | AL035461.3 | 0.52 | 4.96E-27 |
| GGT5 | MSC-AS1 | 0.60 | 4.68E-38 |  | **GSTA2** | LINC02018 | 0.46 | 6.82E-21 |
| NNMT | MSC-AS1 | 0.49 | 5.61E-24 |  | **ACYP1** | AC026333.4 | 0.51 | 1.01E-25 |
| ACYP1 | THUMPD3-AS1 | 0.40 | 3.96E-16 |  |  |  |  |  |

**TableS3. Metabolic lncRNAs that significantly associated with the prognosis of gastric cancer**

| gene | HR | HR.95L | HR.95H | pvalue |
| --- | --- | --- | --- | --- |
| PGM5P4-AS1 | 1.87 | 1.07 | 3.28 | 0.028959 |
| LINC02773 | 2.27 | 1.22 | 4.23 | 0.009497 |
| AC009065.9 | 0.66 | 0.44 | 0.98 | 0.041144 |
| AL356417.2 | 2.15 | 1.41 | 3.29 | 0.000411 |
| AP001189.3 | 1.19 | 1.03 | 1.37 | 0.014903 |
| LINC01140 | 2.90 | 1.29 | 6.53 | 0.010263 |
| SENCR | 1.57 | 1.08 | 2.27 | 0.01759 |
| LINC02544 | 1.11 | 1.03 | 1.20 | 0.006813 |
| MAGI2-AS3 | 1.21 | 1.02 | 1.43 | 0.025782 |
| AL590705.3 | 2.04 | 1.22 | 3.40 | 0.006482 |
| AL139393.3 | 1.12 | 1.03 | 1.23 | 0.011791 |
| MSC-AS1 | 1.42 | 1.07 | 1.89 | 0.016503 |
| SREBF2-AS1 | 0.76 | 0.59 | 0.97 | 0.030119 |
| CYP1B1-AS1 | 3.50 | 1.12 | 10.95 | 0.031727 |
| AP001528.1 | 1.42 | 1.11 | 1.83 | 0.006009 |
| AL139147.1 | 3.76 | 1.50 | 9.45 | 0.004789 |
| AL033527.3 | 0.30 | 0.11 | 0.80 | 0.016464 |
| MAPKAPK5-AS1 | 0.91 | 0.83 | 1.00 | 0.04586 |
| NR2F1-AS1 | 1.34 | 1.01 | 1.77 | 0.041931 |
| AC093278.2 | 1.23 | 1.02 | 1.50 | 0.034275 |
| PSMG3-AS1 | 1.41 | 1.03 | 1.94 | 0.033531 |
| AC110995.1 | 2.05 | 1.35 | 3.11 | 0.000706 |
| AC012615.1 | 0.75 | 0.57 | 0.99 | 0.040108 |
