# Supplementary material for: The value of metabolic LncRNAs in predicting prognosis and immunotherapy efficacy of gastric cancer
Source: Front Oncol. 2023 Jan 4;12:1019909. doi: 10.3389/fonc.2022.1019909 (PMC9845566; doi:10.3389/fonc.2022.1019909)
Supplement: Supplementary file 3 [file DataSheet_3.docx]

**Supplementary Figures**

**
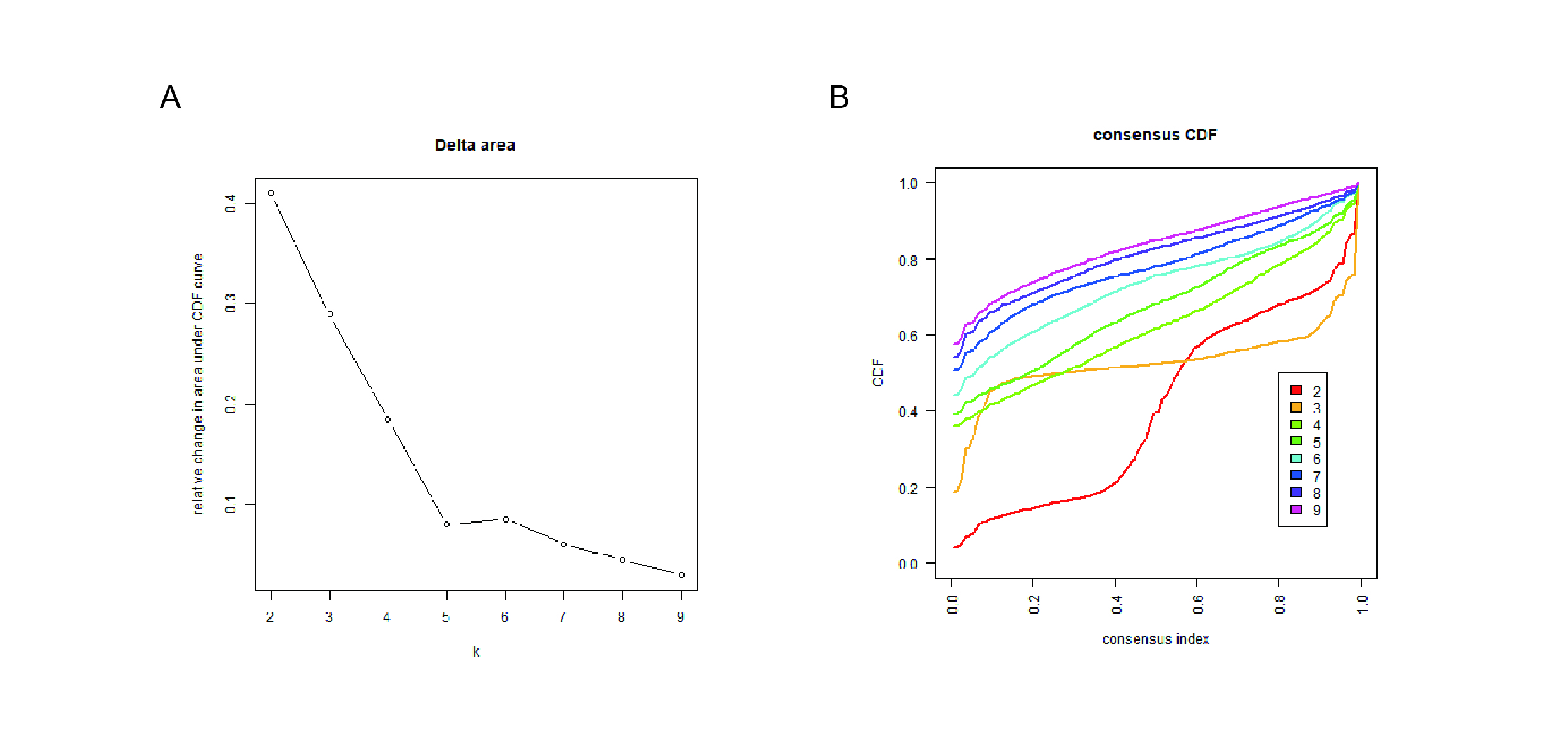
**

**Figure S1.** Consensus clustering of the cumulative distribution function for k = 2–9.


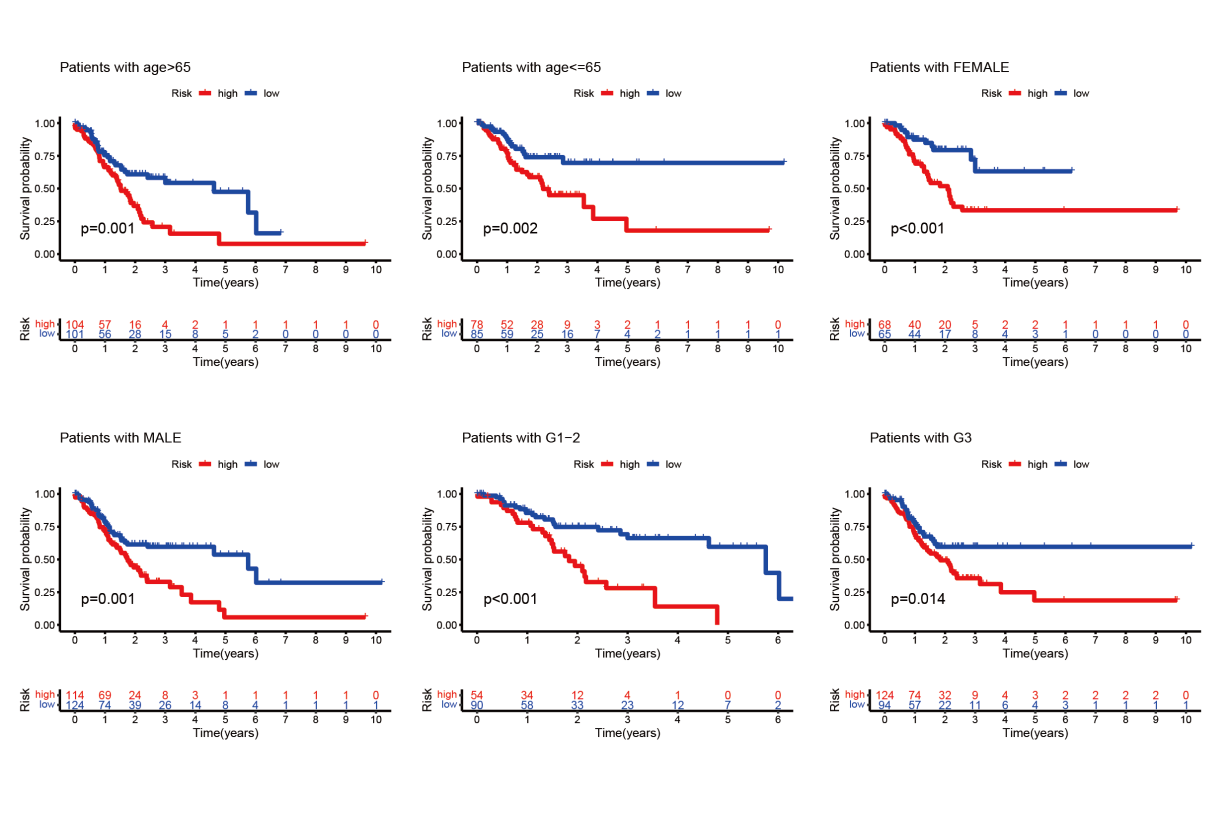


**Figure S2.** Kaplan-Meier curves of OS differences stratified by age, gender and grade between the high- and low-risk groups.


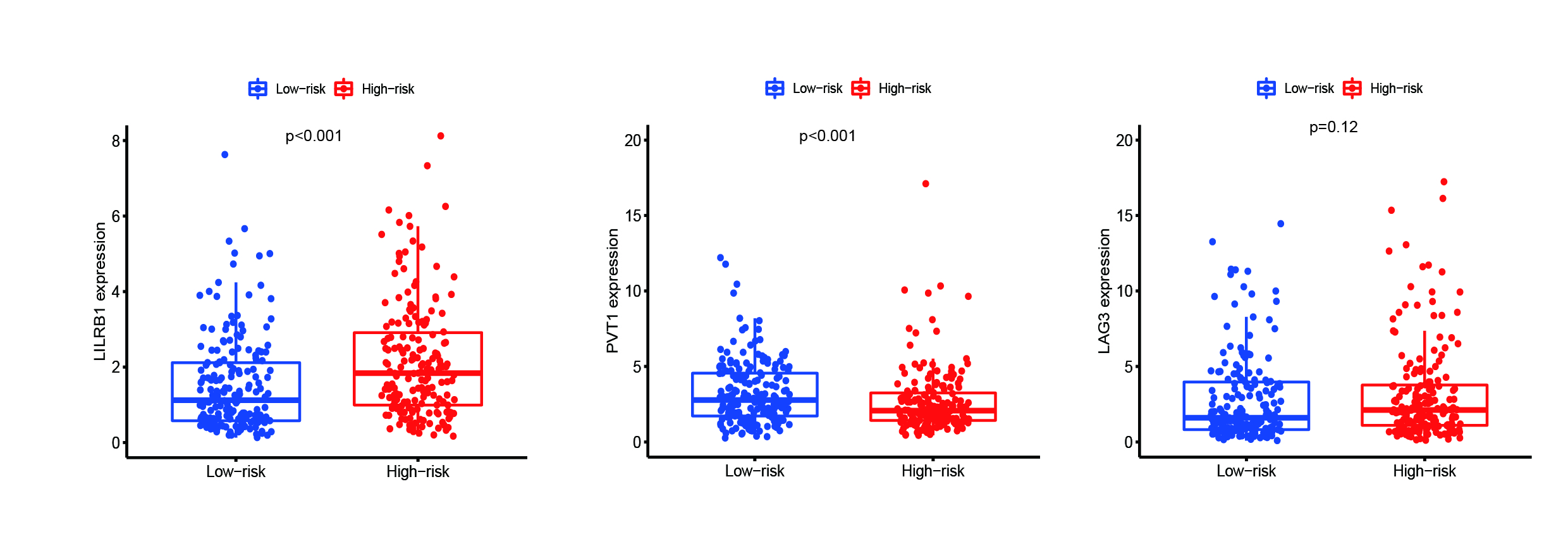
**Figure S3.** The relationship between immune genes or oncogenes and risk scores.


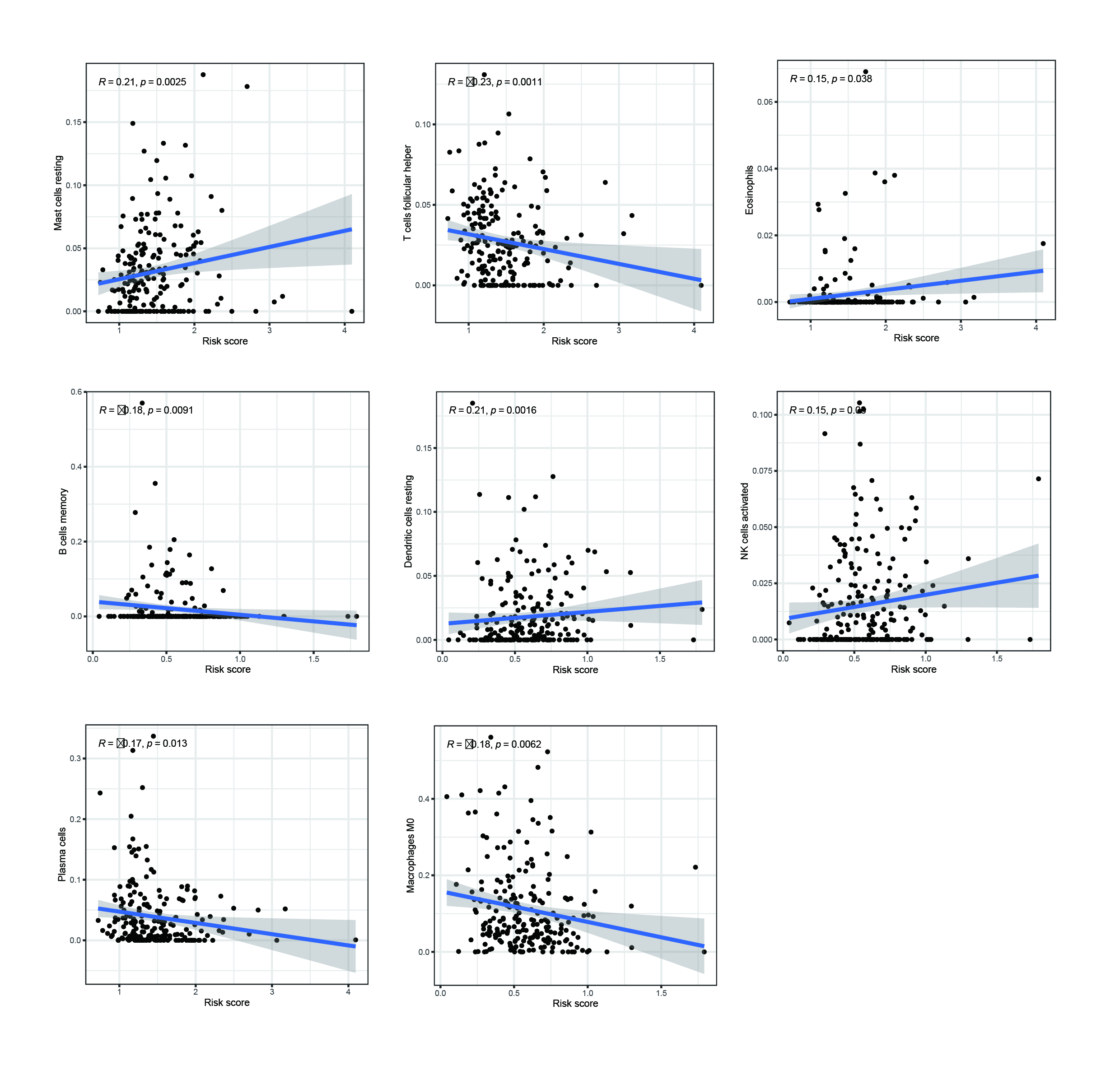


**Figure S4**. The relationship between immune cell types and risk scores.
